# Dried blood spot improves global access to aquaporin‐4‐IgG testing for neuromyelitis optica

**DOI:** 10.1002/acn3.52178

**Published:** 2024-10-15

**Authors:** Nisa Vorasoot, Yahya J. Abdulrahman, Farrah Mateen, James P. Fryer, Vyanka Redenbaugh, Jessica A. Sagen, Abdu K. Musubire, Sarah M. Jenkins, Amy P. Gorsh, John J. Chen, Anastasia Zekeridou, Andrew McKeon, Eoin P. Flanagan, John R. Mills, Sean J. Pittock

**Affiliations:** ^1^ Department of Neurology Mayo Clinic Rochester Minnesota USA; ^2^ Department of Laboratory Medicine and Pathology Mayo Clinic Rochester Minnesota USA; ^3^ Center of MS and Autoimmune Neurology Mayo Clinic Rochester Minnesota USA; ^4^ Division of Neurology, Department of Medicine, Faculty of Medicine Khon Kaen University Khon Kaen Thailand; ^5^ Department of Neurology Massachusetts General Hospital Boston Massachusetts USA; ^6^ Department of Medicine School of Medicine, College of Health Sciences, Makerere University Kampala Uganda; ^7^ Department of Quantitative Health Sciences, Division of Clinical Trials and Biostatistics Mayo Clinic Rochester Minnesota USA; ^8^ Department of Ophthalmology Mayo Clinic Rochester Minnesota USA

## Abstract

**Objective:**

This study aimed to evaluate the diagnostic accuracy of dried blood spot (DBS) compared with conventional serum Aquaporin‐4‐IgG (AQP4‐IgG) testing.

**Methods:**

Prospective multicenter diagnostic study was conducted between April 2018 and October 2023 across medical centers in the United States, Uganda, and the Republic of Guinea. Neuromyelitis optica spectrum disorder (NMOSD) patients and controls collected blood on filter paper cards along with concurrent serum samples. These samples underwent analysis using flow cytometric live‐cell‐based assays (CBA) and enzyme‐linked immunosorbent assay (ELISA) to determine AQP4 serostatus. The accuracy of AQP4‐IgG detection between DBS and serum (gold standard) was compared.

**Results:**

Among 150 participants (47 cases, 103 controls), there was a strong correlation between DBS and serum samples (Spearman's correlation coefficient of 0.82). The AUC was 0.97 (95% CI: 0.92–0.99). AQP4‐IgG detection through DBS showed 87.0% sensitivity (95% CI: 0.74–0.95) and 100% specificity (95% CI: 0.96–1.00) using CBA, and 65.2% sensitivity (95% CI: 0.43–0.84) and 95.2% specificity (95% CI: 0.76–0.99) using ELISA. Serum ELISA demonstrated 69.6% sensitivity (95% CI: 0.47–0.87) and 98.4% specificity (95% CI: 0.91–0.99). The stability of DBS in detecting AQP4‐IgG persisted over 24 months for most cases.

**Interpretation:**

The DBS represents a viable alternative for detecting AQP4‐IgG in resource‐limited settings to diagnose NMOSD, offering high sensitivity and specificity comparable to serum testing. Moreover, DBS has low shipping costs, is easy to administer, and is suitable for point‐of‐care testing.

## Introduction

Neuromyelitis optica spectrum disorder (NMOSD) primarily affects the central nervous system (CNS), characterized by relapsing episodes of severe optic neuritis and myelitis.^1^, ^2^ The discovery of the aquaporin‐4 (AQP4) antibody in 2004 delineated NMOSD as a distinct disorder from multiple sclerosis (MS) and related disorders. However, global awareness of NMOSD has been limited, partly due to the lack of accessible diagnostic antibody testing. In 2023, the World Health Organization (WHO) included three immunosuppressive medicines in the Essential Medicines List, bringing new attention to treatment and accurately classifying the neuroimmunological disorder for appropriate drug selection across countries of varying income levels.

NMOSD represents a model neuroimmunological disorder with a specific disease biomarker and severe disability. NMOSD imposes a prominent disease burden, with up to one‐third of patients facing blindness or paralysis within 5 years from the first attack without proper disease‐specific immunosuppressive treatment.^3^ The overall mortality rate in NMOSD is up to 32%.^4^ The prevalence of NMOSD varies, with estimates ranging from 0.34 to 10 cases per 100,000 in adults and 0.06 to 0.22 per 100,000 in children.^5^ Incidence rates range between 0.039 and 0.73 cases per 100,000 person‐years in adults and between 0.01 and 0.06 cases per 100,000 person‐years in children.^6^

A recent systematic review revealed that the Afro‐Caribbean region has the highest prevalence and incidence estimates of NMOSD, with individuals of African ancestry experiencing the highest burden, while those of White race have the lowest.^5^, ^6^ However, the true global epidemiology of NMOSD remains unknown, primarily due to limited access to antibody testing and neurological care in lower‐income countries.^7^

The presence of AQP4‐IgG in serum serves as a hallmark of NMOSD, as these pathogenic antibodies bind to astrocytic AQP4, initiating complement‐mediated neuronal tissue damage.^1^ Detecting AQP4‐IgG enables precise differentiation and diagnosis of NMOSD from other CNS conditions. Various methodologies have been employed for detecting AQP4‐IgG, including cell‐based assays (CBA) using AQP4 transfected HEK 293 cells, enzyme‐linked immunosorbent assays (ELISA), and tissue indirect immunofluorescence assays (IFA).^8^, ^9^, ^10^, ^11^, ^12^, ^13^, ^14^ To date, live CBA has demonstrated the highest sensitivity (0.76, 95% CI: 0.67–0.82) and specificity (0.99, 95% CI: 0.97–0.99),^15^ and CBA was recommended as the detection method of choice by the 2015 diagnostic criteria committee.^2^ Despite these advancements, the global availability of serum antibody testing for AQP4 remains limited due to the requirement for advanced techniques in specialized laboratories. Only half of African and Eastern Mediterranean countries have access to AQP4‐IgG testing, with only 15% of World Bank‐defined low‐income countries having access to these antibody tests.^7^ A prompt and accurate diagnosis of NMOSD is crucial as it enables the initiation of effective disease‐modifying therapies that can reduce the risk of relapses and improve patient outcomes.

Conventional antibody testing relies on serum samples, which can pose logistical challenges in terms of collection, storage, and transportation. Dried blood spots (DBS) have emerged as a potential alternative due to their ease of collection, stability, and cost‐effectiveness.^16^ Consequently, this study aimed to evaluate the diagnostic utility and accuracy of DBS as an alternative to conventional serum AQP4‐IgG testing.

## Methods

### Study design

This was a prospective multicenter study conducted between April 2018 and October 2023, predominantly involving two medical centers located in the United States of America and Uganda. Additionally, two samples from the Republic of Guinea were included for analysis. The study protocol received approval from the institutional review boards of all participating centers, and written informed consent was obtained from all patients.

### Participants

#### Inclusion and exclusion criteria

For cases, inclusion criteria were individuals aged 18 years or older with a clinical diagnosis of NMOSD and/or serologically confirmed diagnosis of AQP4‐IgG seropositive NMOSD, according to the International Panel for NMO Diagnosis (IPND) Criteria 2015.^2^ For controls, we included individuals aged 18 years or older with a diagnosis of other neurological diseases and a confirmed AQP4‐IgG seronegative status.

#### Recruitment

Participants were prospectively recruited through in‐person visits at outpatient neurology clinics at Mayo Clinic, Rochester, MN, USA, Mulago National Referral Hospital, Kampala, Uganda, and Ignace Deen Hospital, Conakry, the Republic of Guinea. Additionally, recruitment was conducted remotely by mailing information to patients enrolled in the Mayo Clinic Center for Multiple Sclerosis and Autoimmune Neurology Biorepository.

### Samples collection and transportation

**Figure 1:**
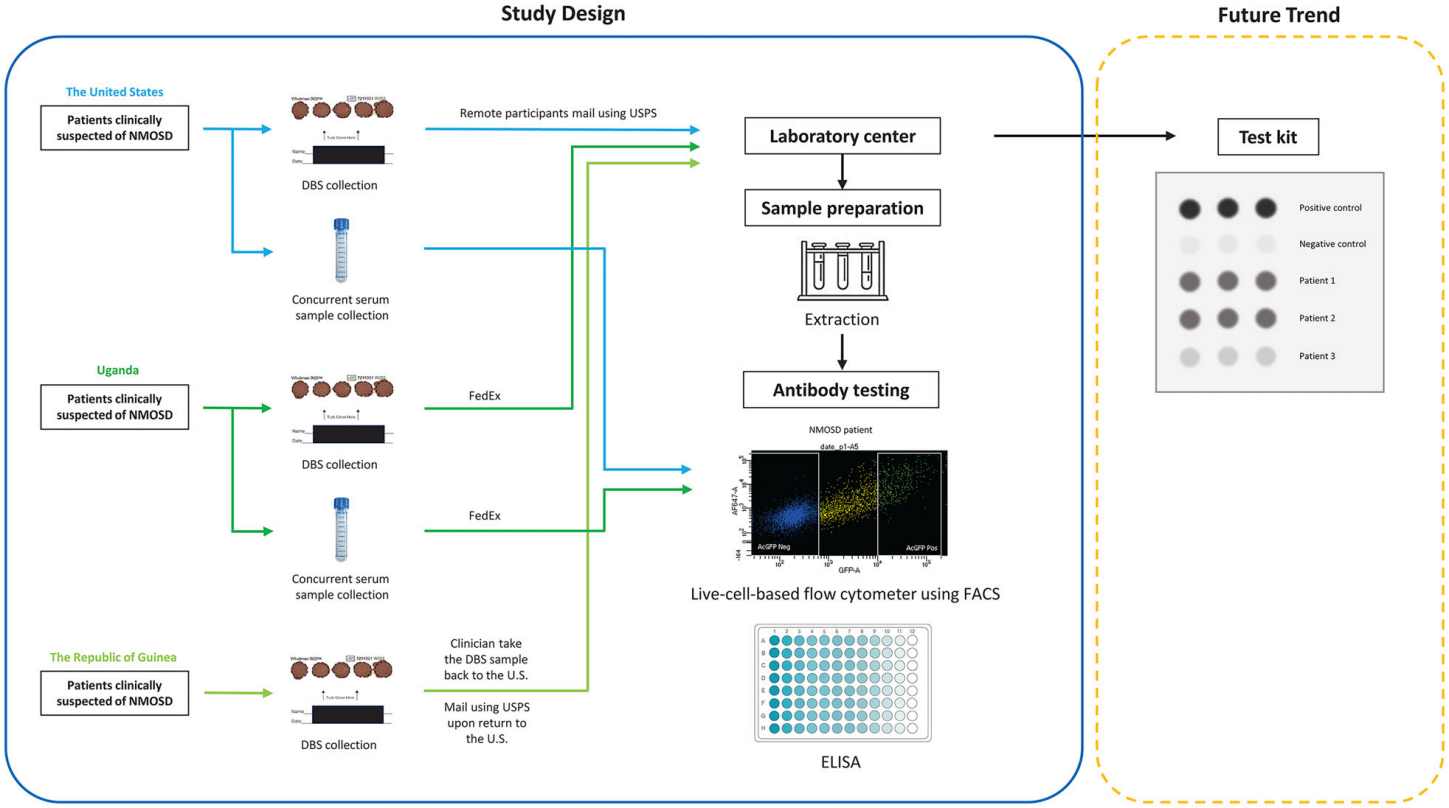
Workflow of the study: patients suspected of NMOSD (remote participants) in the United States received Whatman® 903 Protein Saver Cards by mail. They used these cards for blood spot collection and then returned their samples using pre‐paid business reply envelopes through regular post (non‐temperature controlled). DBS and concurrent serum samples from Uganda were collected on the same day, the serum samples were stored at −80°C, while the DBS samples were stored at room temperature (RT) until testing. Both DBS samples and simultaneous serum samples were mailed to the United States using a FedEx Express Pak. In the Republic of Guinea, a clinician collected DBS samples, stored them at RT, and mailed them using regular post upon return to the United States. All these collected samples were sent to the Mayo Clinic Neuroimmunology Laboratory for analysis. The DBS samples were extracted and subjected to AQP4‐IgG testing using a live‐cell‐based flow cytometer with FACS and ELISA, alongside the evaluation of serum samples. In the future, there is potential for developing a kit that employs an immunodot assay for detecting AQP4‐IgG. Notably, Patient 1 and Patient 2 demonstrated positive results, while Patient 3 tested negative. Abbreviations: NMOSD, neuromyelitis optica spectrum disorder; DBS, dried blood spot; FACS, fluorescence‐activated cell sorting; ELISA, enzyme‐linked immunosorbent assay; AF647, Alexafluor 647; AcGFP, Aequorea coerulescens green fluorescent protein; GFP, green fluorescent protein.

**Figure 2:**
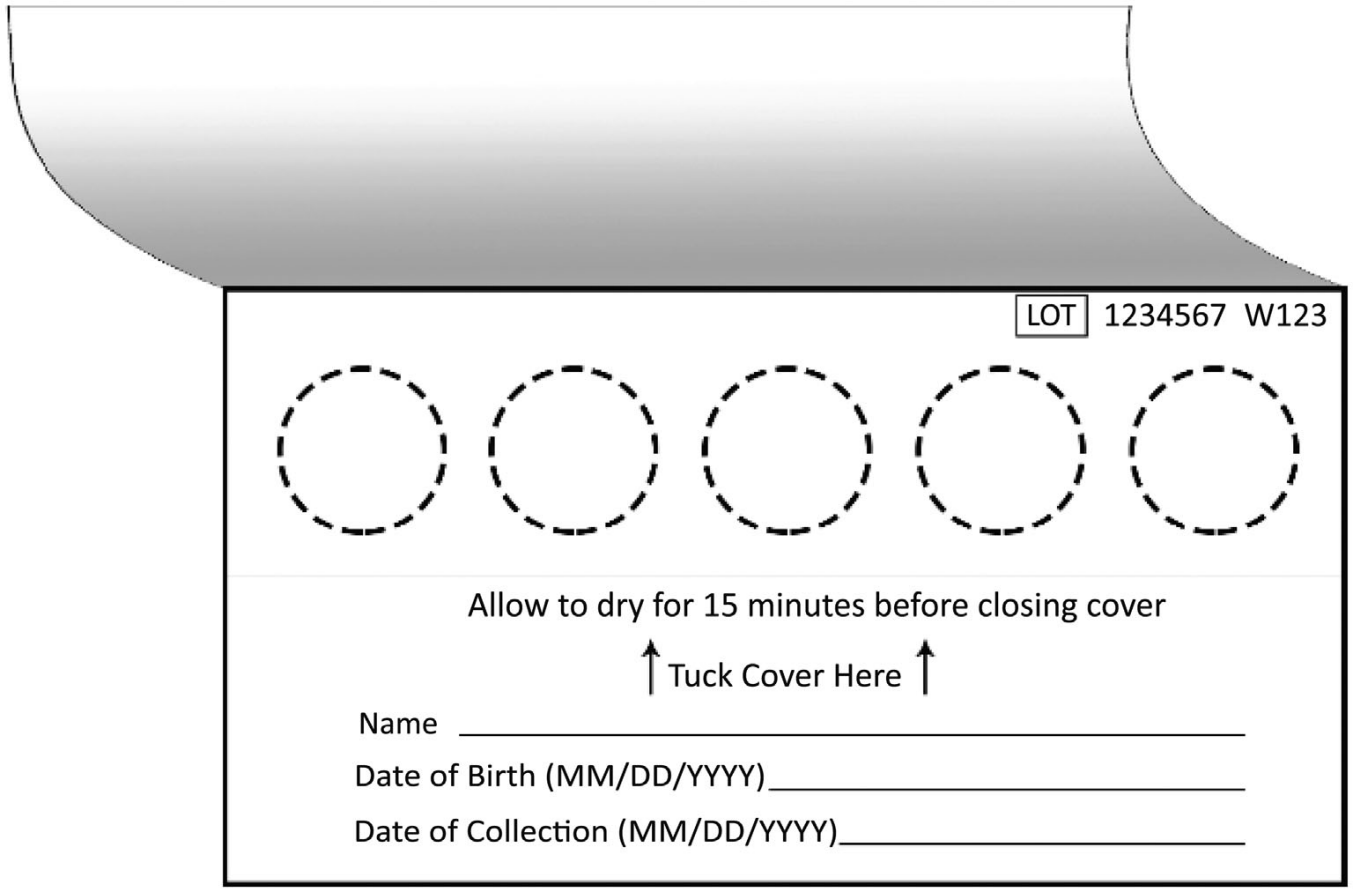
Whatman® 903 Protein Saver Cards.

Participants utilized 1.5 mm contact‐activated lancets to obtain blood samples from their fingers, yielding approximately 1 mL of blood (Fig. 1). Subsequently, they placed three to five blood spots into predefined circles on Whatman® 903 Protein Saver Cards (Fig. 2). The cards were allowed to air dry completely for approximately 3 h and were then sealed in plastic biohazard bags. These DBS cards were stored at room temperature (RT) until laboratory analysis.

For transporting DBS samples, remote participants in the United States received study materials and instructions by mail and returned their samples using pre‐paid business reply envelopes through regular post (non‐temperature controlled). In the Republic of Guinea, a clinician collected DBS samples, stored them at RT, and mailed them using regular post upon return to the United States. The DBS samples from Uganda were collected by laboratory technologists, stored at RT until testing, and then shipped to the United States using a FedEx Express Pak. The shipping process took approximately 10 days, mainly due to issues encountered during scheduling a pick‐up at remote locations.

Concurrently, corresponding serum samples were collected from both Mayo Clinic and Uganda patients. The Uganda serum samples were kept frozen and shipped to Mayo Clinic via FedEx using dry ice frozen boxes. Upon arrival at the Mayo Clinic Neuroimmunology Laboratory, they were stored at −80°C until testing. Serum samples from Mayo Clinic patients were also stored at −80°C until testing. Both DBS and corresponding serum samples were masked before being sent in batches to the Mayo Clinic Neuroimmunology Laboratory for analysis.

### Samples preparation

We utilized a puncher to obtain a 13 mm circular punch of the DBS, corresponding to approximately 80 μL of whole blood. Subsequently, the punched DBS circle was placed in 200 μL of live‐cell binding buffer (LCBB), consisting of phosphate‐buffered saline (PBS) with 2% bovine serum albumin (BSA), 10% normal goat serum, 15 mmol/L ethylenediaminetetraacetic acid (EDTA), and 0.05% sodium azide. The prepared samples were stored overnight at 4°C to facilitate the elution of AQP4‐IgG into the solution. Meanwhile, the serum sample was thawed and kept at 4°C the day before the analysis.

### Antibody testing

A flow cytometric live CBA, utilizing fluorescence‐activated cell sorting (FACS), was employed to detect and quantify the levels of AQP4 antibodies in both the DBS and concurrent serum samples as per the Mayo Clinic Neuroimmunology Laboratory protocol.^13^ The mixture, comprising the filter paper and 200 μL of LCBB, was subjected to centrifugation, following which the supernatant was removed and heated at 56°C for 35 min to inactivate complement proteins. The inactivated samples were then added to live HEK 293 cells that had been transiently transfected with the human AQP4 M1‐isoform, co‐expressing a non‐linked green fluorescent protein (GFP) (pIRES2‐AcGFP hAQP4/M1 plasmid DNA). Each sample was run in duplicate at a 1:5 dilution. To detect AQP4‐IgG, a Pan‐IgG Fc‐specific secondary antibody (Alexa Fluor 647‐conjugated goat anti‐human IgG, Southern Biotech catalog number 2040‐31) was used. The quantification of the autoantibody was accomplished by calculating the IgG binding index (IBI), which is defined as the ratio of the median fluorescence intensity (MFI) of transfected cells to that of non‐transfected cells:

$$\mathrm{IgG}\;\text{Binding Index}\;\left(\mathrm{IBI}\right)=\frac{\mathrm{MFI}\;\mathrm{GFP}+\text{Population}}{\mathrm{MFI}\;\mathrm{GFP}-\text{Population}}$$

An AQP4‐IBI value equal to or greater than 2.0 was considered a positive result. We conducted follow‐up AQP4‐IgG tests for all seropositive cases using the same DBS at approximately 0, 6, 12, and 24 months to evaluate the stability of DBS over time.

Additionally, we utilized the AQP4 ELISA assay (Kronus, Star, ID, USA) to detect the levels of AQP4 antibodies in both DBS and concurrent serum samples. Samples were tested according to the manufacturer's instructions. Reagents were allowed to stand at RT for at least 30 min prior to use. Then, 50 μL of each patient's serum, calibrators, and positive and negative control samples provided by the manufacturer were pipetted into a 96‐well plate coated with recombinant human M1‐AQP4. One well was left empty for the blank. Additionally, 25 μL of biotinylated AQP4 was pipetted into each well (except the blank). After incubating for 2 h at RT on a shaker, the well contents were discarded by briskly inverting the frame of strip wells over a suitable receptacle. The wells were washed three times manually, and the inverted wells were gently tapped on a clean, dry absorbent surface to remove excess wash. Subsequently, 100 μL of diluted streptavidin–peroxidase was pipetted into each well (except the blank). After incubating for 20 min at RT on a shaker, the wash steps were repeated. Pure water was used for the final wash step before tapping the wells dry. Next, 100 μL of tetramethyl benzidine was pipetted into each well (including the blank) and incubated for 20 min in the dark at RT without shaking. Finally, 50 μL of stop solution was added to each well (including the blank) and the plates were shaken for 5 sec. The absorbance of each well was read at 405 nm using an ELISA plate reader, blanked against a well containing 100 μL of TMB substrate and 50 μL of stop solution only. A calibration curve was established by plotting the calibrator concentration on the *x*‐axis (log scale) against the absorbance of the calibrators on the *y*‐axis (linear scale). The arbitrary AQP4 autoantibody units (AU) in patient sera were then read off the calibration curve. According to the manufacturer's instructions, samples with values equal to or greater than 5 AU/mL were considered positive.^9^, ^10^

### Sample size calculation

The sample size was calculated based on the desired precision for estimating the area under the receiver operating characteristic curve (AUC), along with the proportion of NMOSD from a prior study involving a point‐of‐care, filter paper‐based test for serum AQP4‐IgG.^17^ With 150 samples, assuming an NMOSD proportion of 30% and AUC of 0.90, we can estimate the AUC with a 95% confidence interval width of 12.6 percentage points.

### Statistical analysis

Statistical analyses were conducted using BlueSky Statistics Version 10.3.1 and R package version 8.81. Baseline characteristics were presented as means (standard deviation; SD) and medians (interquartile ranges [IQR]). The correlation between AQP4‐IBI values obtained from DBS and serum samples using CBA was estimated with adjusted Spearman's correlation coefficient (partial correlation was used to adjust for the time difference between DBS collection and testing for cases in which exact date of collection was known). Positive correlations were interpreted as follows: 0 to 0.19, very weak positive; 0.2 to 0.39, weak positive; 0.4 to 0.59, moderate positive; 0.6 to 0.79, strong positive; 0.8 to 1.0, very strong positive. These AQP4‐IBI values were graphically depicted on the log scale due to skewness. To evaluate the overall performance of DBS in AQP4‐IgG detection, a receiver operating characteristic (ROC) curve was generated (including the earliest DBS obtained for those who had multiple tests). The AUC was calculated, along with its corresponding 95% confidence interval (CI). Diagnostic properties based on a AQP4‐IBI value cutoff of ≥2.0 for DBS, encompassing sensitivity and specificity were summarized (positive predictive values (PPV), negative predictive value (NPV), and likelihood ratio values were not shown due to their high influence on the overall proportion with the disease). Sensitivity and specificity of ELISA were also analyzed. Sensitivity and specificity levels were categorized as follows: below 70% as low, 70–85% as moderate, and above 85% as high. The Youden index was employed to determine the best cutoff point.

## Results

### Demographic data of participants

Among the 150 participants, 47 had a confirmed diagnosis of NMOSD, with 103 participants serving as controls. Demographic information for the participants is presented in Table 1. Out of the 47 individuals diagnosed with NMOSD, 46 were serologically confirmed with AQP4‐IgG seropositive NMOSD, while one was clinically diagnosed with NMOSD. The mean age at disease onset was 55.87 (SD: 15.71) years. The majority of these cases were female (87.0%), and most were of White race (72.3%). The mean disease duration from onset to blood collection was 10.33 (SD: 9.09) years. Within the group of 103 controls, the diagnoses included myelin oligodendrocyte glycoprotein antibody‐associated disease (MOGAD) 56 (77.8%), idiopathic optic neuritis 3 (4.2%), transverse myelitis 2 (2.8%), stiff person syndrome 2 (2.8%), MS 1 (1.4%), paraneoplastic cerebellar degeneration 1 (1.4%), collapsin response mediator protein 5 (CRMP5) 1 (1.4%), anti‐Ma2 encephalitis 1 (1.4%), glial fibrillary acidic protein (GFAP) encephalitis 1 (1.4%), chronic lymphocytic inflammation with pontine perivascular enhancement responsive to steroids (CLIPPERS) 1 (1.4%), cryptococcosis 1 (1.4%), encephalopathy 1 (1.4%), and lumbar spondylosis 1 (1.4%).

**Table 1:** Participants’ demographic data.

	NMOSD cases (*N* = 47)	Controls (*N* = 103)	Total (*N* = 150)
Country, *N* (%)			
USA	39 (83.0%)	85 (82.5%)	124 (82.7%)
Uganda	7 (14.9%)	17 (16.5%)	24 (16.0%)
The Republic of Guinea	1 (2.1%)	1 (1.0%)	2 (1.3%)
Age at onset (years), mean (SD)	55.87 (15.71)	47.29 (14.29)	50.45 (15.34)
Female, *N* (%)	40 (87.0%)	61 (68.5%)	101 (74.8%)
White	34 (72.3%)	77 (74.8%)	111 (74.0%)
Black	11 (23.4%)	18 (17.5%)	29 (19.3%)
Asian	2 (4.3%)	2 (1.9%)	4 (2.7%)
Unknown	0 (0.0%)	6 (5.8%)	6 (4.0%)
Ethnicity, *N* (%)			
Hispanic	1 (2.1%)	0 (0.0%)	1 (0.7%)
Not Hispanic or Latino	45 (95.7%)	97 (94.2%)	142 (94.7%)
Unknown	1 (2.1%)	6 (5.8%)	7 (4.7%)
Disease duration from onset to blood collection (years), mean (SD)	10.33 (9.09)	6.36 (5.21)	9.34 (8.43)
Duration from blood collection to laboratory analysis (days), median (IQR)	38.50 (20.25, 89.25)	42.00 (22.00, 150.75)	42.00 (21.00, 94.75)

NMOSD, neuromyelitis optica spectrum disorder; SD, standard deviation; IQR, interquartile ranges.

### Correlation between AQP4‐IBI values obtained from DBS and serum samples using CBA

**Figure 3:**
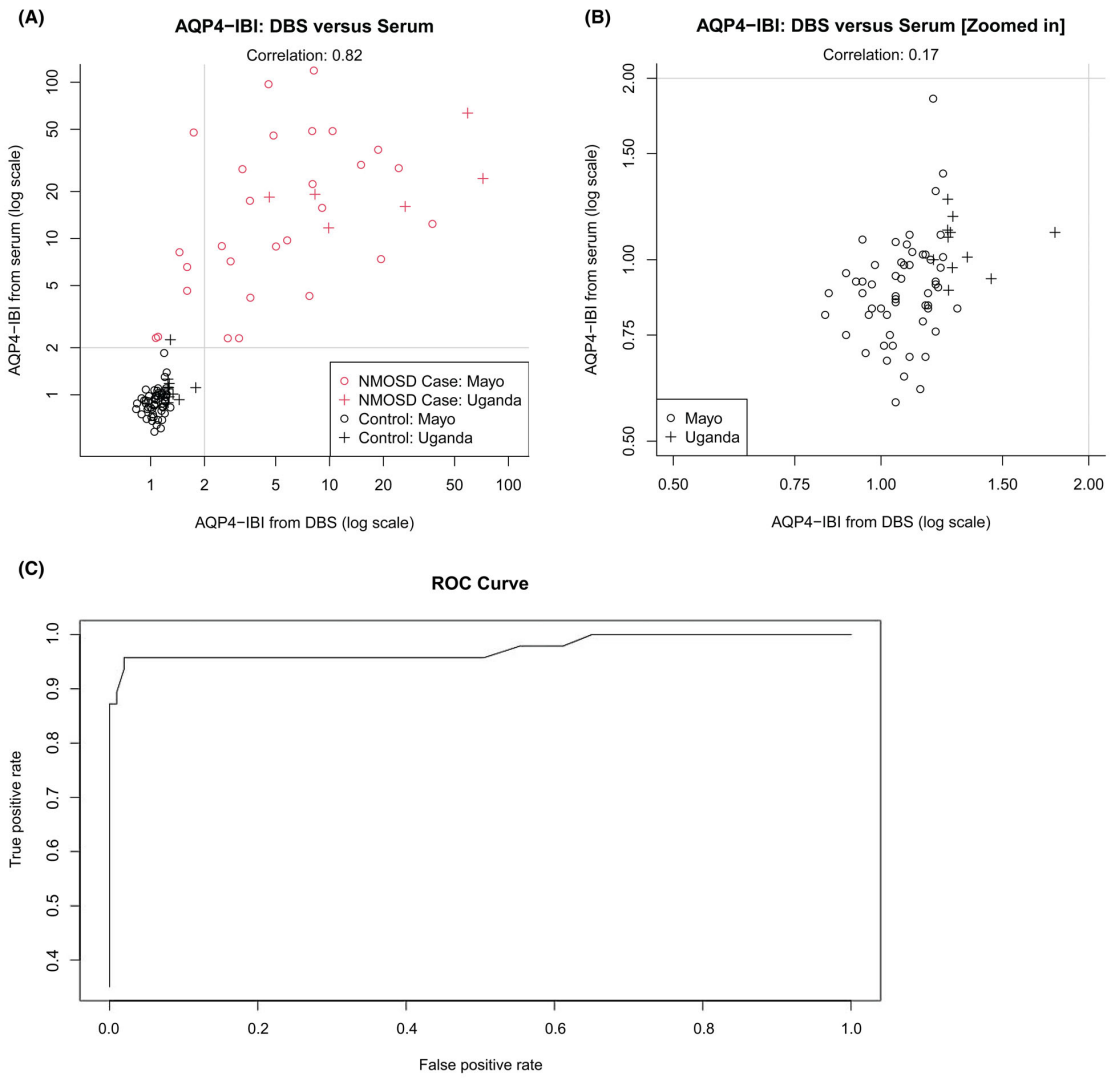
Scatter plot demonstrates a correlation of 0.82 between AQP4‐IBI from DBS and serum, displayed in both full scale (A) and a zoomed‐in scale based on the subset where both DBS and serum IBI values are ≤2, revealing a correlation of 0.17 (B). Additionally, it includes subgroup analysis focusing on patients from the United States and Uganda. In the plot, NMOSD cases are indicated in red, while negative controls are represented in black. Reference lines at 2 indicate the positive cutoff results. The overall performance of DBS in AQP4‐IgG detection, indicated by the AUC of the ROC curve, was 0.97 (95% CI: 0.92–0.99) (C). Abbreviations: ROC curve, receiver operating characteristic curve; AQP4‐IBI, aquaporin‐4‐IgG binding index; DBS, dried blood spot; NMOSD, neuromyelitis optica spectrum disorder.

A very strong correlation was observed between AQP4‐IBI values obtained from DBS and serum samples using CBA, indicated by a very strong Spearman's correlation coefficient of 0.82. Subgroup analysis performed on patients from the United States showed a strong Spearman's correlation coefficient of 0.79, while Uganda patients exhibited a moderate Spearman's correlation coefficient of 0.60. However, subgroup analysis could not be carried out for two patients from the Republic of Guinea due to absence of serum AQP4‐IBI results. Figure 3A, B illustrates this correlation through a scatter plot of the two tests and the subgroup analysis.

The mean AQP4‐IBI obtained from serum was 8.76 (SD: 18.92), whereas from DBS, it was 3.98 (SD: 8.96). Based on an IBI cutoff of 2, which is the established serum IBI cutoff, among 46 cases positive for AQP4‐IgG through serum testing, 40 (87.0%) were also positive through DBS, while 6 (13.0%) with low IBI tested negative via DBS. For the 102 control samples negative for AQP4‐IgG through serum, all 102 (100%) were also negative when tested via DBS. Notably, one control from Uganda exhibited AQP4‐IBI values obtained from serum of 2.25 and from DBS of 1.29, the final diagnosis was cryptococcosis.

### Diagnostic accuracy and determination of the optimal cutoff for AQP4‐IgG seropositivity

The overall performance of DBS in AQP4‐IgG detection using CBA, indicated by the AUC of the ROC curve, was 0.97 (95% CI: 0.92–0.99), as demonstrated in Figure 3C. The diagnostic properties of DBS in AQP4‐IgG detection across various cutoff's are summarized in Table 2. Based on the Youden index, the optimal cutoff for AQP4‐IBI values obtained from DBS was identified as 1.45. However, we established a conservative cutoff of 2.00, as it provided a sensitivity of 80% and specificity of 100%. Based on a cutoff of ≥2, AQP4‐IgG detection through DBS yielded a sensitivity of 87.0% (95% CI: 0.74–0.95) and a specificity of 100% (95% CI: 0.96–1.00), as presented in Table 3. ELISA, using a cutoff of ≥5 AU/mL, provided a sensitivity of 69.6% (95% CI: 0.47–0.87) and a specificity of 98.4% (95% CI: 0.91–0.99) for serum samples, and a sensitivity of 65.2% (95% CI: 0.43–0.84) and a specificity of 95.2% (95% CI: 0.76–0.99) for DBS samples (Table 4).

**Table 2:** Performance of DBS in AQP4‐IgG detection using CBA at various cutoffs.

Cutoff	Sensitivity (%)	Specificity (%)	Youden index
1.40	95.7	96.1	0.92
1.45^1^	95.7	98.1	0.94
1.60	93.6	98.1	0.92
1.80	87.2	99.0	0.86
2.00	87.2	100.0	0.87

DBS, dried blood spot; AQP4‐IgG, aquaporin‐4‐IgG; CBA, cell‐based assays.

^1^ Optimal cutoff, per Youden Index.

**Table 3:** Performance of DBS in AQP4‐IgG detection using CBA based on a cutoff of ≥2.

DBS AQP4‐IgG testing by CBA results	Standard serum AQP4‐IgG testing by CBA results			Sensitivity, % (95% CI)	Specificity, % (95% CI)
	AQP4‐IgG seropositive cases	AQP4‐IgG seronegative controls	Total		
Positive	40	0	40	87.0% (0.74–0.95)	100.0% (0.96–1.00)
Negative	6	102	108		
Total	46	102	148		

DBS, dried blood spot; AQP4‐IgG, aquaporin‐4‐IgG; CBA, cell‐based assays; CI, confidence interval.

**Table 4:** Performance of DBS and serum in AQP4‐IgG detection using ELISA based on a cutoff of ≥5 AU/mL.

AQP4‐IgG testing by ELISA	Sensitivity, % (95% CI)	Specificity, % (95% CI)
DBS	65.2% (0.43–0.84)	95.2% (0.76–0.99)
Serum	69.6% (0.47–0.87)	98.4% (0.91–0.99)

DBS, dried blood spot; AQP4‐IgG, aquaporin‐4‐IgG; ELISA, enzyme‐linked immunosorbent assay; CI, confidence interval.

### Stability over time of DBS

**Figure 4:**
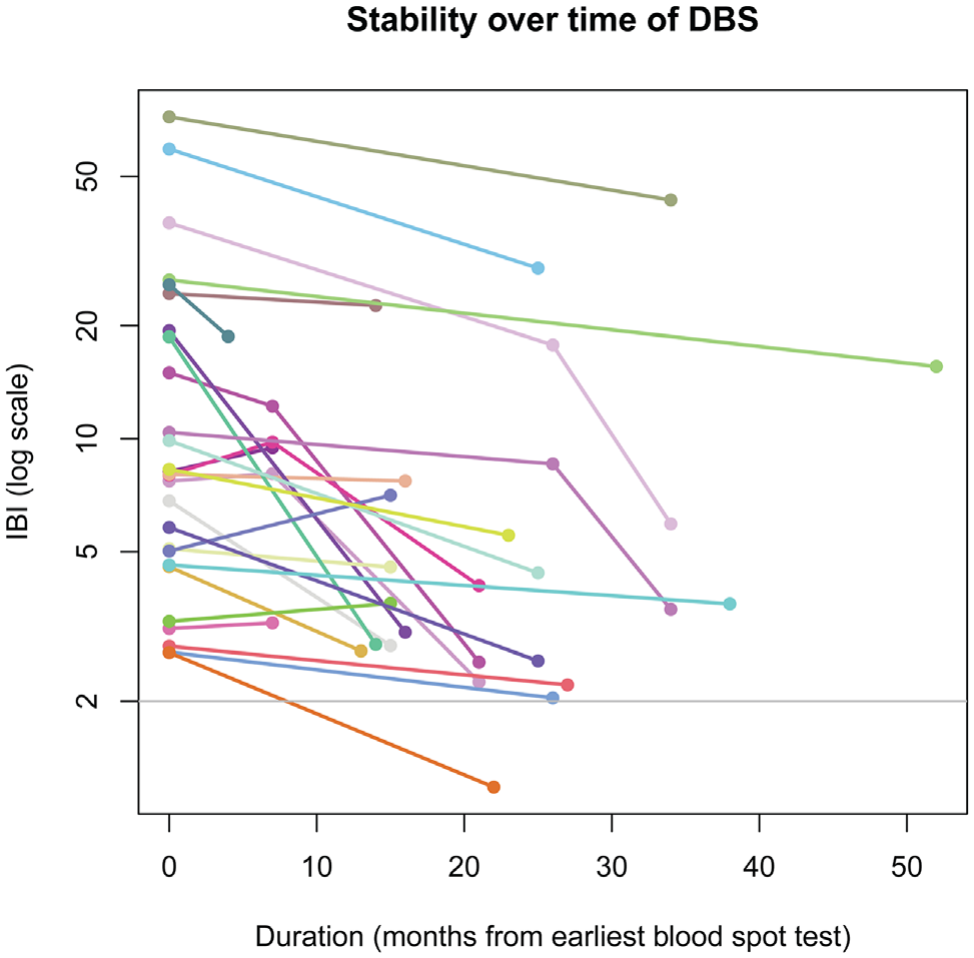
Stability over time of DBS in the detection of AQP4‐IgG was assessed in seropositive cases by evaluating AQP4‐IBI values approximately at 6, 12, and 24 months utilizing the same DBS. This revealed the persistence of a positive status throughout the 24‐month period for most cases, except for one patient with an initially low IBI (initial IBI of 2.69), who transitioned from a positive to a negative status. Abbreviations: DBS, dried blood spot; IBI, IgG binding index.

The long‐term stability of DBS in detecting AQP4‐IgG using CBA was assessed in seropositive cases by evaluating AQP4‐IBI values at approximately 6, 12, and 24 months, using the same DBS. This analysis revealed the persistence of a positive status throughout the 24‐month period for most cases, except for one patient with an initially low IBI (initial IBI of 2.69), who transitioned from a positive to a negative status (Fig. 4). We found that the stability of DBS over time was positively associated with the titer, which correlates with disease activity.

## Discussion

In our prospective multicenter study, we evaluated the diagnostic accuracy of DBS as a potential alternative to conventional serum AQP4‐IgG testing. The findings revealed high sensitivity and specificity of DBS in detecting AQP4‐IgG using CBA, estimated at 87.0% (95% CI: 0.74–0.95) and 100% (95% CI: 0.96–1.00), respectively. Since ELISA is accessible in some geographic regions, we also assessed the diagnostic accuracy of DBS in detecting AQP4‐IgG using ELISA, revealing a sensitivity of 65.2% (95% CI: 0.43–0.84) and a specificity of 95.2% (95% CI: 0.76–0.99). Although the sensitivity and specificity for DBS in detecting AQP4‐IgG using ELISA are lower than for CBA, they are comparable to serum samples. ELISA provided a sensitivity of 69.6% (95% CI: 0.47–0.87) and a specificity of 98.4% (95% CI: 0.91–0.99) for serum samples. These results indicate the potential of DBS as a practical alternative to traditional serum testing methods. Our study included a large sample size (150 participants) from diverse settings across multiple centers, encompassing participants from low‐income countries where such a test would have pragmatic utility.

Rice *et al*. conducted a pilot study investigating AQP4‐IgG testing through DBS, demonstrating promising outcomes with a sensitivity of 80% and specificity of 93%.^17^ The study had limitations, including a small sample size of 40 participants, absence of simultaneous standard serum antibody testing, use of fixed CBA with transfected M23‐AQP4‐GFP HEK293 cells for antibody detection, and determination of AQP4 serostatus through fluorescent microscopy. Additionally, the lack of a definitive quantitative cutoff led to subjective result interpretation. Our study utilized live CBA with transfected M1‐AQP4‐GFP HEK293 cells and determined AQP4 serostatus through FACS, which is a CLIA (Clinical Laboratory Improvement Amendments)‐approved test. This approach exhibited improved performance compared with the previous method. Additionally, we conducted retesting to demonstrate stability over time. Furthermore, we evaluated the optimal cutoff for AQP4‐IgG seropositivity and established it for AQP4‐IBI values obtained via DBS at 2.00. This approach aimed to provide a clearer and more objective basis for interpreting results in AQP4‐IgG testing using DBS.

Collecting blood samples requires expertise, typically from a phlebotomist. Moreover, the storage and transportation of blood and serum specimens involve significant costs and challenges, particularly in regions where maintaining proper conditions, such as temperature control, sterile techniques, and preventing contamination, can be difficult. However, the emergence of DBS technology has provided a valuable alternative to conventional blood or serum sampling methods. DBS collection is simpler, minimally invasive, and cost‐effective, requiring a smaller volume of blood compared with traditional venipuncture, minimizing the risk of bacterial contamination and hemolysis, and allowing preservation for extended periods with minimal degradation of the analytes at RT.^18^ Notably, blood samples on filter paper in triple packaging, as employed in DBS, are not categorized as dangerous goods.^19^ This characteristic makes transporting these filter paper tests more feasible compared with transporting serum in microtubes. Primarily, DBS has found widespread use in screening neonates for congenital and inherited metabolic disorders.^20^ Its initial applications included serological testing for the diagnosis of various infectious diseases.^21^, ^22^ The versatility and practical advantages of DBS have positioned it as a valuable tool in diagnostic testing, especially in situations where traditional blood sample collection methods encounter logistical challenges. Our study obtained samples from Uganda and the Republic of Guinea, where antibody testing is unavailable, demonstrating the feasibility of accessing antibody testing from a high‐income country through DBS collection in resource‐limited settings. Moreover, DBS can serve as a viable transfer method for ELISA, offering potential cost savings on shipping, and expediting turnaround times when performed locally.

Fu *et al*. conducted a study comparing the immunodot assay to the gold standard CBA in detecting AQP4‐IgG. The immunodot assay exhibited an overall sensitivity of 99.4% (95% CI: 97.8–99.9%) and a specificity of 99.2% (95% CI: 98.0–99.8%).^23^ This assay provides a practical and cost‐effective alternative for detecting AQP4‐IgG without requiring specialized laboratories. As a result, it holds significant implications for affordable testing in low‐income countries. The utilization of DBS as a transportation method instead of serum transfer and employing the immunodot assay as a replacement for live CBA, could potentially serve as an approach in regions with limited resources in the future (Fig. 1).

In 2018, a global survey highlighted that in high‐income nations, 70–100% of patients can access treatment without encountering overwhelming health expenditures. In stark contrast, in low‐income countries, this capability is limited to less than 10% of patients. Even in low‐income countries, there is a great divide between the rural areas where majority live and the urban areas where some degree of neurological services exist. DBS can be of use in this divide with samples carried from rural to urban areas for analysis. Moreover, in most low‐income nations, patients bear the entire cost of NMOSD diagnostics and care without any public assistance.^7^ In July 2023, the WHO updated the Model Lists of Essential Medicines, incorporating rituximab for the indication of MS, potentially expanding its availability for NMOSD.^24^ This move is supported by class 1 evidence showing its high efficacy in preventing attacks in NMOSD,^25^ along with its cost advantages over other novel attack‐prevention medications for this condition. The potential accessibility of rituximab in low‐income countries, and the expansion of more cost‐effective biosimilars, holds promise for increasing its availability for NMOSD treatment, particularly in lower‐income countries. Plasma exchange (PE) is considered for use as rescue or as first‐line therapy for NMOSD, and early PE leads to improved neurological outcomes.^26^, ^27^ Establishing a confirmed diagnosis is important for initiating PE. It underscores the crucial need to expand accessibility to AQP4‐IgG diagnostic testing.

Our study has several limitations. Firstly, due to the unavailability of antibody testing and the inability to transport serum specimens from the Republic of Guinea, we lack serological information for patients from this region. Secondly, variations in blood spot sizes on some DBS filter papers led to inconsistent blood sample volumes, potentially impacting the assay's sensitivity and contributing to the occurrence of false‐negative results observed during our study. Thirdly, some serum and DBS samples were not collected on the same date, which may have influenced the test results. Future studies with larger sample sizes and varying clinical settings would be beneficial for confirming these findings. Additionally, including a study arm with serum samples shipped at RT could potentially reduce shipping costs and offer a viable option for resource‐limited regions.

## Conclusions

Our study established the reliability and diagnostic potential of DBS for detecting AQP4‐IgG. The findings demonstrated that AQP4‐IgG detection using DBS exhibits both high sensitivity and high specificity, almost comparable to the current gold standard serum testing. This method proved feasibility in resource‐limited regions and within the United States, performed by patients themselves. Moreover, our approach indicates the potential for expanding the use of DBS to test for other neurological antibodies or neuroinflammatory biomarkers.

## Author Contributions

NV, YJA, JRM, and SJP contributed to the conception and design of the study. NV, YJA, FM, JPF, VR, JAS, AKM, SMJ, APG, JJC, AZ, AM, EPF, JRM, and SJP contributed to the acquisition and analysis of data. NV, YJA, FM, EPF, JRM, and SJP contributed to drafting the text or preparing the figures.

## Conflicts of Interest

JPF, AZ, AM, EPF, JRM, and SJP are working in the Mayo Clinic neuroimmunology laboratory clinical service that offers AQP4‐IgG testing and may offer AQP4‐IgG testing using DBS in the future but none of the authors receive personal income from these tests. NV, YJA, FM, VR, JAS, AKM, SMJ, APG, and JJC report no disclosures.

## Data Availability

Anonymized data used for this study will be made available from the corresponding author upon reasonable request.
